# Mathematical modelling of antibiotic interaction on evolution of antibiotic resistance: an analytical approach

**DOI:** 10.7717/peerj.16917

**Published:** 2024-02-26

**Authors:** Ramin Nashebi, Murat Sari, Seyfullah Enes Kotil

**Affiliations:** 1 Department of Mathematics, Yildiz Technical University, Istanbul, Turkey; 2 Department of Mathematical Engineering, Istanbul Technical University, Istanbul, Turkey; 3 Department of Biophysics, Bahcesehir University, Istanbul, Turkey; 4 Department of Molecular Biology and Genetics, Bogazici University, Istanbul, Turkey

**Keywords:** Antibiotic, Antibiotic interaction, Differential equation, Equilibrium solutions

## Abstract

**Background:**

The emergence and spread of antibiotic-resistant pathogens have led to the exploration of antibiotic combinations to enhance clinical effectiveness and counter resistance development. Synergistic and antagonistic interactions between antibiotics can intensify or diminish the combined therapy’s impact. Moreover, these interactions can evolve as bacteria transition from wildtype to mutant (resistant) strains. Experimental studies have shown that the antagonistically interacting antibiotics against wildtype bacteria slow down the evolution of resistance. Interestingly, other studies have shown that antibiotics that interact antagonistically against mutants accelerate resistance. However, it is unclear if the beneficial effect of antagonism in the wildtype bacteria is more critical than the detrimental effect of antagonism in the mutants. This study aims to illuminate the importance of antibiotic interactions against wildtype bacteria and mutants on the deacceleration of antimicrobial resistance.

**Methods:**

To address this, we developed and analyzed a mathematical model that explores the population dynamics of wildtype and mutant bacteria under the influence of interacting antibiotics. The model investigates the relationship between synergistic and antagonistic antibiotic interactions with respect to the growth rate of mutant bacteria acquiring resistance. Stability analysis was conducted for equilibrium points representing bacteria-free conditions, all-mutant scenarios, and coexistence of both types. Numerical simulations corroborated the analytical findings, illustrating the temporal dynamics of wildtype and mutant bacteria under different combination therapies.

**Results:**

Our analysis provides analytical clarification and numerical validation that antibiotic interactions against wildtype bacteria exert a more significant effect on reducing the rate of resistance development than interactions against mutants. Specifically, our findings highlight the crucial role of antagonistic antibiotic interactions against wildtype bacteria in slowing the growth rate of resistant mutants. In contrast, antagonistic interactions against mutants only marginally affect resistance evolution and may even accelerate it.

**Conclusion:**

Our results emphasize the importance of considering the nature of antibiotic interactions against wildtype bacteria rather than mutants when aiming to slow down the acquisition of antibiotic resistance.

## Introduction

Bacterial antibiotic resistance poses a complex and increasingly significant public health issue on a global scale (Thompson, 2022; World Health Organization, 2023). Infections caused by resistant bacteria present greater challenges in treatment compared to those caused by non-resistant bacteria, often leading to prolonged hospital stays of over 13 days (Touat et al., 2021; Mauldin et al., 2010; Ventola, 2015), increased healthcare costs (Mauldin et al., 2010; Llor & Bjerrum, 2014), and a 46% higher mortality rate than diseases such as HIV/AIDS and Malaria (Thompson, 2022). Key challenges associated with antibiotic resistance include the rapid evolution of resistance (Aminov & Mackie, 2007), inadequate diagnostics, the scarcity of new antibiotics (Boucher et al., 2013; So & Shah, 2014), the misuse and overuse of antibiotics (Paterson et al., 2016), and *de novo* development of resistance during treatment (Davies & Davies, 2010).

Antibiotics can either synergistically enhance or antagonistically reduce the effects of combined therapy. Additive interactions refer to antibiotics that act independently without influencing each other’s effects (Gullberg et al., 2011). Synergistic interactions occur when two antibiotics intensify each other’s inhibitory effects, effectively eliminating susceptible bacteria as they would individually with greater effect than would be seen with an additive reaction (Michel et al., 2008; Torella et al., 2010; Yeh et al., 2009). Conversely, antagonistic interactions diminishes the inhibitory effects, resulting in less effective suppression or elimination of susceptible bacteria compared to individual use an additive interaction (Michel et al., 2008; Torella et al., 2010; Yeh et al., 2009). Combination therapy involving more than two medications can exhibit additive, antagonistic, and synergistic effects (Pimenta et al., 2014).

Minimum inhibitory concentration (MIC) is a microbiological parameter that aids in the selection of suitable antibiotics for therapy (Kowalska-Krochmal & Dudek-Wicher, 2021). It indicates the lowest concentration of an antimicrobial agent that can prevent visible growth of microorganisms after overnight incubation (Kowalska-Krochmal & Dudek-Wicher, 2021; Andrews, 2001). Antibiotics with lower MIC values are more potent in killing microorganisms per dose.

Bacteria have the ability to develop multidrug resistance (MDR) against antibiotics from the same or different classes, employing various mechanisms (Peterson & Kaur, 2018; Thitiananpakorn et al., 2020; Colclough et al., 2019; Sanders et al., 1984; Colclough et al., 2019; Woodford & Ellington, 2007). MDR emergence and dissemination are primarily driven by chromosomal gene mutation and horizontal gene transfer (HGT) (Techitnutsarut & Chamchod, 2021; Richardson, 2017; Sun et al., 2019; Munita & Arias, 2016; Blair et al., 2015; Revitt-Mills & Robinson, 2020). In many infections, high mutation rates lead to resistance against individual drugs (Fauci, 2003; Bhusal, Shiohira & Yamane, 2005; Edwards & Biagini, 2006; White et al., 1999). Consequently, the use of multidrug treatment has been proposed to enhance therapeutic efficacy by maximizing the eradication rate of mutant strains (Michel et al., 2008; Barriere, 1991).

*Staphylococcus aureus* is a major concern due to its multidrug resistance (Michel et al., 2008; Moran et al., 2006; Hiramatsu et al., 2014). While horizontal gene transfer is a primary source of resistance in *S. aureus*, vertically acquired resistance through spontaneous mutations is also worrisome, leading to the use of combination therapies to prevent their development (Michel et al., 2008). This bacterium exhibits resistance to β-lactam antibiotics, as well as other classes such as aminoglycosides, tetracyclines, and fluoroquinolones, which limits the available antibacterial treatment options against infections caused by these bacteria (Thitiananpakorn et al., 2020).

In treatments requiring continuous drug exposure for the desired therapeutic effect, antibiotics are often administered at a constant rate (McCarthy & Avent, 2020; Eichenberger, Fowler & Holland, 2020). This typically involves intravenous infusion to maintain therapeutic blood levels over an extended period, particularly in the treatment of severe infections caused by *Staphylococcus aureus* (Eichenberger, Fowler & Holland, 2020), such as sepsis or endocarditis, where a specific antibiotic concentration must be maintained in the bloodstream for effective bacterial eradication. The duration of treatment for this disease typically ranges from 7 to 10 days (Taylor & Unakal, 2022).

The clinical objective is to eliminate as many infectious bacteria as possible, inhibit their growth to allow the immune system to take control, and prevent antibiotic resistance (Hegreness et al., 2008; Michel et al., 2008). In clinical practice, susceptible infections are typically treated with a single antibiotic, although synergistic drug combinations may be used for increased potency. However, *in vitro* studies have explored various combination therapies to minimize the development of resistance. Both empirical (Chait, Craney & Kishony, 2007; Hegreness et al., 2008; Michel et al., 2008; Torella et al., 2010; Pena-Miller et al., 2013) and modeling (Torella et al., 2010; Techitnutsarut & Chamchod, 2021; Volkova et al., 2012; Campbell & Chao, 2014; Arya et al., 2021) studies have investigated how the evolution of antibiotic resistance is influenced by the synergistic and antagonistic interactions of antibiotics in the context of combination therapy.

Several studies have demonstrated that the use of antagonistically interacting antibiotics in treating wildtype bacteria results in a slower development of resistance compared to synergistic antibiotics (Hegreness et al., 2008; Chait, Craney & Kishony, 2007; Yeh et al., 2009; Torella et al., 2010). Another study revealed that wildtype bacteria treated with synergistic drugs eventually developed resistance, leading to a switch from synergistic to antagonistic interaction (Pena-Miller et al., 2013). Consequently, resistance acquisition appears to accelerate when drugs act antagonistically on mutants and decelerate when they interact antagonistically on wildtype bacteria. However, it remains unclear whether the beneficial effect of antagonistic interaction in wildtype bacteria outweighs the detrimental impact on mutants. Thus, this study aims to elucidate the significance of antagonistic interactions between antibiotics in relation to mutant and wildtype bacteria, focusing on the deceleration of antimicrobial resistance.

This study aims to fill the existing research gap by formulating and analyzing a mathematical model that describes the population dynamics of wildtype and mutant bacteria under different antibiotic combinations. The synergism or antagonism of the combination is the other independent parameter of our model. Specifically, we investigate the growth rate of mutants that acquire resistance to two selected antibiotics, which interacts synergistically or antagonistically (for the wildtype or the mutant). To simplify the analyses, we have chosen to work with two antibiotics. The novelty of this work lies in our ability to analytically explore the relationship between antibiotic interactions for both wildtype and mutant bacteria in the context of antibiotic resistance. Moreover, our model provides an explicit equation for the growth rate of mutants as a function of antibiotic interaction levels, spanning from antagonism to synergism.

## Model and methods

### Model formulation

Portions of this text were previously published as part of a preprint (Nashebi, Sari & Kotil, 2022). In our study, we focus on modeling the scenario of multidrug treatment against the *Staphylococcus aureus* bacteria in an individual. The population sizes of wildtype and mutant bacteria at time *t* are denoted as *S(t)* and *R(t)*, respectively. We assume that bacterial growth follows a logistic model with a carrying capacity *K*. The birth rate of wildtype bacteria is represented by *β*_*s*_, and the birth rate of mutant bacteria is denoted as *β*_*r*_. It is important to note that specific mutations that confer resistance to chemical control incur a fitness cost, which can result in reduced reproductive capacity or competitive ability (Alavez-Ramírez et al., 2007). We quantify this fitness cost as a reduction in the reproduction rate of the mutant strain, leading to *β*_*r*_ *≤ β*_*s*_. The natural death rates for wildtype and mutant bacteria are represented as *μ*_*s*_ and *μ*_*r*_, respectively. Additionally, both bacterial types can also die due to the action of antibiotics. In this study, we consider two types of antibiotics: (1) antibiotic agent *M*, which kills both wildtype and mutant bacteria, (2) antibiotic agent *N*, which also kills both wildtype and mutant bacteria. The antibiotic *M* kills wildtype and mutant bacteria with rates ${\alpha _{11}}$ and ${\alpha _{21}}$, while the antibiotic *N* affects them with rates ${\alpha _{12}}$ and ${\alpha _{22}}$, respectively. Moreover, these antibiotics interact synergistically and antagonistically to kill wildtype and mutant bacteria. To account for the combined effect of antibiotics *M* and *N* in killing both wildtype and mutant bacteria, we adopt a density-dependent approach based on the work of Ibargüen-Mondragón et al. (2014). This allows us to explore the relationship between the pharmacodynamics of the antibiotics and the population dynamics of wildtype and mutant bacteria when exposed to these antibiotics. The model is described as follows:

(1a) $$\overline {{X_s}} = \left( {\overline {{\alpha _{11}}} {C_1} + \overline {{\alpha _{12}}} {C_2} + {\lambda _1}\overline {{\alpha _{11}}} \; \overline {{\alpha _{12}}} {C_1}{C_2}} \right)$$

(1b) $$\overline {{X_r}} = \left( {\overline {{\alpha _{21}}} {C_1} + \overline {{\alpha _{22}}} {C_2} + {\lambda _2}\overline {{\alpha _{21}}} \; \overline {{\alpha _{22}}} {C_1}{C_2}} \right)$$

where

(2a) $$\overline {{\alpha _{11}}} = \displaystyle{{E_{max}^{M,S}} \over {IC_{50}^{M,S}}}$$

(2b) $$\overline {\; {\alpha _{12}}} = \displaystyle{{E_{max}^{N,S}} \over {IC_{50}^{N,S}}}$$

and

(3a) $$\overline {{\alpha _{21}}} = \displaystyle{{E_{max}^{M,R}} \over {IC_{50}^{M,R}}}$$

(3b) $$\overline {\; {\alpha _{22}}} = \displaystyle{{E_{max}^{N,R}} \over {IC_{50}^{N,R}}}$$

where, *E*^*M,S*^_*max*_ and *E*^*N,S*^_*max*_ represent the maximal killing rates of antibiotics *M* and *N* of wildtype bacteria, *E*^*M,R*^_*max*_ and *E*^*N,R*^_*max*_ represent the maximal killing rates of antibiotics *M* and *N* of mutant bacteria. *IC*^*M,S*^_*50*_ and *IC*^*N,S*^_*50*_ signify the half-maximal inhibitory concentration of the antibiotics $M$ and *N* for wildtype bacteria. *IC*^*M,S*^_*50*_ and *IC*^*N,S*^_*50*_ denote the half-maximal inhibitory concentration of the antibiotics *M* and *N* for mutant bacteria. Multiplication of Eqs. (2a), (2b) and (3a), (3b) give Emax model (Salahudeen & Nishtala, 2017; Holford, 2017) which quantify the relationship between concentration and effect of antibiotics.

The parameters *λ*_*1*_ and *λ*_*2*_ represent the interaction strengths for wildtype and mutant bacteria, respectively. These parameters have a range of −1.5 to 1.5 (−1.5 ≤ *λ*_*1*_, *λ*_*2*_ ≤ 1.5) (Torella et al., 2010). Negative values indicate antagonistic interactions between the antibiotics, while positive values indicate synergistic interactions.

Bacteria have the potential to acquire resistance to both antibiotic agents through mutation. The concentrations of antibiotics *M* and *N* are denoted as *C*_*1*_*(t)* and *C*_*2*_*(t)*, respectively. We assume that these two antibiotics belong to the same class and have the same inhibitory effect. Furthermore, we assume that both antibiotics bind to the same target, allowing bacteria to develop resistance to both antibiotics through a single mutation event. The acquisition of resistance by mutant bacteria from wildtype bacteria is modeled by the terms *q*_*1*_*C*_*1*_*(t)S(t)* and *q*_*2*_*C*_*2*_*(t)S(t)*, where *q*_*1*_ and *q*_*2*_ represent the mutation rates of wildtype bacteria when exposed to antibiotics, respectively.

To maintain a constant concentration of antibiotics *M* and *N*, they are supplied at a constant rate *θ*_*1*_ and *θ*_*2*_, respectively. Antibiotics are removed from the system at a constant per capita rate *μ*_*1*_ and *μ*_*2*_, respectively.

Under the assumptions revealed above, we obtain the following system of differential equations:

(4a) $$\displaystyle{{dS} \over {dt}} = {\beta _s}S\left( {1 - \displaystyle{{S + R} \over K}} \right) - \left( {{q_1}{C_1} + {q_2}{C_2}} \right)S - \left( {\overline {{X_s}} + {\mu _s}} \right)S$$

(4b) $$\displaystyle{{dR} \over {dt}} = {\beta _r}R\left( {1 - \displaystyle{{S + R} \over K}} \right) + \left( {{q_1}{C_1} + {q_2}{C_2}} \right)S - \left( {\overline {{X_r}} + {\mu _r}} \right)R$$

(4c) $$\displaystyle{{d{C_1}} \over {dt}} = {\theta _1} - {\mu _1}{C_1}\;$$

(4d) $$\displaystyle{{d{C_2}} \over {dt}} = {\theta _2} - {\mu _2}{C_2}$$

with the consideration of following change of variable:

$s = \displaystyle{S \over K},r = {R \over K}, {c_1} = {{{C_1}} \over {{\theta _1}/{\mu _1}}}, {c_2} = \displaystyle{{{C_2}} \over {{\theta _2}/{\mu _2}}}$

the non-dimensionalized system (4) can be rewritten as:

(5a) $$\displaystyle{{ds} \over {dt}} = {\beta _s}s\left( {1 - \left( {s + r} \right)} \right) - ({q_1}{c_1} + {q_2}{c_2})s - \left( {{X_s} + {\mu _s}} \right)s$$

(5b) $$\displaystyle{{dr} \over {dt}} = {\beta _r}r\left( {1 - \left( {s + r} \right)} \right) + ({q_1}{c_1} + {q_2}{c_2})s - \left( {{X_r} + {\mu _r}} \right)r$$

(5c) $$\displaystyle{{d{c_1}} \over {dt}} = {\mu _1} - {\mu _1}{c_1}$$

(5d) $$\displaystyle{{d{c_2}} \over {dt}} = {\mu _2} - {\mu _2}{c_2}$$

where

(6a) $${X_s} = \left( {{\alpha _{11}}{c_1} + {\alpha _{12}}{c_2} + {\lambda _1}{\alpha _{11}}\; {\alpha _{12}}{c_1}{c_2}} \right)$$

(6b) $${X_r} = \left( {{\alpha _{21}}{c_1} + {\alpha _{22}}{c_2} + {\lambda _2}{\alpha _{21}}\; {\alpha _{22}}{c_1}{c_2}} \right).$$

and

${\alpha _{1i}} = \overline {{\alpha _{1i}}} \left( {{\theta _i}/{\mu _i}} \right)$

${\alpha _{2i}} = \overline {{\alpha _{2i}}} \left( {{\theta _i}/{\mu _i}} \right) ,i = 1,2$

The region of biological interest of system (5) is given by

(7) $${\Omega } = \left\{ {\left( {s,r,{c_1},{c_2}} \right)\; \epsilon \; R_ + ^4\; :\; 0 \le s,r,{c_1},{c_2} \le 1,\; \; \; 0 \le s + r \le 1\; } \right\}.$$

The set Ω defined in (7) is positively invariant for the system (5) (Ibargüen-Mondragón et al., 2019). Consequently, the system (5) is well-posed because solutions with initial conditions in *Ω* remain there for all *t ≥ 0*.

### Qualitative analysis of the model

In this part, we will analyze the solutions of system (5) which include infection-free, all-mutant, and coexistence of wildtype and mutant bacteria equilibrium-points. We will then investigate the stability conditions of these solutions based on the antibiotic interaction parameters (λ1 and λ2) for wildtype and mutant bacteria.

### Equilibrium solutions

The model (5) always contains the infection-free equilibrium *P*_*0*_ *= (0,0,1,1)* in Ω. This equilibrium point represent state where both wildtype and mutant bacteria are eliminated under combination therapy. If *R*_*r*_ *> 1*, *P*_*1*_ *= (0, (R*_*r*_ − *1)/R*_*r*_*,1,1)* is an all-mutant equilibrium in *Ω* where

(8) $${R_r} = \displaystyle{{{\beta _r}} \over {\left( {{\alpha _{21}}{\alpha _{22}}{\lambda _2} + \; {\alpha _{21}} + \; \; {\alpha _{22}}} \right) + {\mu _r}}}.$$

This equilibrium point represent state where only mutant bacteria persist under combination therapy. When *R*_*s*_ *> 1* and *R*_*s*_ *> R*_*r*_ in addition to *P*_*0*_, and *P*_*1*_ there exists a coexistence of wildtype and mutant bacteria equilibrium in *Ω*, *P*_*2*_ *( $\bar s$, $\bar r$,1,1)* where

(9) $${R_s} = \displaystyle{{{\beta _s}} \over {m + \left( {{\alpha _{11}} + {\alpha _{12}} + {\lambda _1}{\alpha _{11}}\; {\alpha _{12}}} \right) + {\mu _s}}},$$

(10) $$\bar r = \displaystyle{{m\left( {\displaystyle{{{R_s} - 1} \over {{R_s}}}} \right)} \over {{\beta _r}\left( {\displaystyle{1 \over {{R_r}}} - \displaystyle{1 \over {{R_s}}}} \right) + m}},$$

and

(11) $$\bar s = \displaystyle{{{R_s} - 1} \over {{R_s}}} - r.$$

This equilibrium point represent state where both wildtype and mutant bacteria persist under combination therapy. The derivation of equilibrium points has been given in Supplemental Information.

Based on the traditional definition of the basic reproduction number, this quantity

(12) $${N_r} = \displaystyle{{{\beta _r}} \over {{\mu _r}}},$$

This parameter is construed as the product of the mutant bacteria’s reproduction rate (*β*_*r*_) and their average lifespan (*1/μ*_*r*_). It signifies the count of bacteria generated by a mutant bacterium throughout its typical lifetime. Likewise,

(13) $${N_s} = \displaystyle{{{\beta _s}} \over {{\mu _s}}}$$

It is understood as the quantity of bacteria generated by a wildtype bacterium during its average lifespan. Conversely, Rs, as defined in Eq. (9), is redefined as

(14) $${R_s} = \displaystyle{{{\mu _s}} \over {m + \left( {{\alpha _{11}} + {\alpha _{12}} + {\lambda _1}{\alpha _{11}}\; {\alpha _{12}}} \right) + {\mu _s}}}{N_s}.$$

Since

(15) $$\displaystyle{{{\mu _s}} \over {m + \left( {{\alpha _{11}} + {\alpha _{12}} + {\lambda _1}{\alpha _{11}}\; {\alpha _{12}}} \right) + {\mu _s}}}$$

specify the proportion of wildtype bacteria that haven’t undergone spontaneous mutations and remain unaffected by antibiotics. Therefore, *R*_*s*_ represents the count of bacteria produced by this fraction of wildtype bacteria that hasn’t undergone spontaneous mutations and remains unaffected by the combination therapy. Similarly, *R*_*r*_ as defined in Eq. (8) is reformulated as

(16) $${R_r} = \displaystyle{{{\mu _r}} \over {\left( {{\alpha _{21}} + {\alpha _{22}} + {\alpha _{21}}{\alpha _{22}}{\lambda _2}\; \; } \right) + {\mu _r}}}{N_r}$$

This represents the count of bacteria produced by the fraction of mutant bacteria that evade the effects of combination therapy.

The results demonstrate the following: (a) when the average bacteria count produced by the fraction of mutant bacteria evading the antibiotic combination effect exceeds one (*R*_*r*_* > 1*), the population of mutant bacteria will endure, (b) if the average bacteria count produced by the fraction of wildtype bacteria without mutations, evading the antibiotics’ combination effect, is greater than one (*R*_*s*_* > 1*), and the average bacteria count produced by the fraction of mutant bacteria exceeds one, both susceptible and resistant bacteria will persist.

### Stability of equilibria points

In this section, we determine the local asymptotic stability of the equilibrium solutions of the system (5). Linearization of the system (5) around point *P* is given by:

(17) $${\vec x^{\prime}} = J\left( P \right)\vec x$$

where

(18) $$\vec x = {\left( {s,r,{c_1},{c_2}} \right)^T}$$

and the matrix *J* evaluated at *P* is:

(19) $$J\left( P \right) = \left[ {\matrix{ {{j_{11}}\left( P \right)} & { - {\beta _s}s} & { - \left( {{c_2}{\lambda _1}{\alpha _{11}}{\alpha _{12}} + {\alpha _{11}}} \right)s} & { - \left( {{c_1}{\lambda _1}{\alpha _{11}}{\alpha _{12}} + {\alpha _{12}}} \right)s} \cr { - {\beta _r}r + m} & {{j_{22}}\left( P \right)} & { - \left( {{c_2}{\lambda _2}{\alpha _{21}}{\alpha _{22}} + {\alpha _{21}}} \right)r} & { - \left( {{c_1}{\lambda _2}{\alpha _{21}}{\alpha _{22}} + {\alpha _{22}}} \right)r} \cr 0 & 0 & { - {\mu _1}} & 0 \cr 0 & 0 & 0 & { - {\mu _2}} \cr } } \right]$$

with

(20a) $${j_{11}}\left( p \right) = {\beta _s}\left( {1 - \left( {s + r} \right)} \right) - {\beta _s}s - m - \left( {\left( {{\alpha _{11}}{c_1} + {\alpha _{12}}{c_2} + {\lambda _1}{\alpha _{11}}{\alpha _{12}}{c_1}{c_2}} \right) + {\mu _s}} \right)$$

(20b) $${j_{22}}\left( p \right) = {\beta _r}\left( {1 - \left( {s + r} \right)} \right) - {\beta _r}r - \left( {\left( {{\alpha _{21}}{c_1} + {\alpha _{22}}{c_2} + {\lambda _2}{\alpha _{21}}\; {\alpha _{22}}{c_1}{c_2}} \right) + {\mu _r}} \right).$$

By evaluating the Eq. (19) Jacobian *J* in *P*_*0*_, *P*_*1*_ *and P*_*2*_ (see Supplemental Information) we obtain that, firstly, if *R*_*s*_ *< 1* and *R*_*r*_ *< 1*, then the infection-free equilibrium *P*_*0*_ is locally and asymptotically stable in Ω. If *R*_*s*_ *> 1* or *R*_*r*_ *> 1*, then *P*_*0*_ is unstable. Since *α*_*11*_, *α*_*12*_, *μ*_*s*_, *and β*_*s*_ are positive; there are three conditions for *R*_*s*_ < 1 if *λ*_*1*_ > 0, *λ*_*1*_ < 0, or *λ*_*1*_ = 0. If *λ*_*1*_ > 0, *λ*_*1*_ < 0 the necessary condition for *R*_*s*_ < 1 is:

(21) $${\beta _s} - {\mu _s} - m < {\alpha _{11}} + {\alpha _{12}} + {\lambda _1}{\alpha _{11}}\; {\alpha _{12}}$$

and if *λ*_*1*_ = 0, the necessary condition is:

(22) $${\beta _s} - {\mu _s} - m < {\alpha _{11}} + {\alpha _{12}}.$$

This implies that when antibiotics combination eliminates the wildtype bacteria, and prohibit the proliferation of mutants, in this case, both bacteria die out. Secondly, If *R*_*r*_ *> R*_*s*_ and *R*_*r*_ *> 1*, then the equilibrium *P*_*1*_ is locally and asymptotically stable in *Ω*. If *R*_*r*_ *< R*_*s*_ or *R*_*r*_ *<1*, then *P*_*1*_ is unstable. Since *α*_*21*_, *α*_*22*_, *μ*_*r*_, *and β*_*r*_ are positive, there are three conditions for *R*_*r*_ > 1, if *λ*_*2*_ > 0, *λ*_*2*_ < 0, or *λ*_*2*_ = 0. If *λ*_*2*_ > 0, *λ*_*2*_ < 0 the necessary condition for *R*_*r*_ < 1 is:

(23) $${\beta _r} - {\mu _r} > {\alpha _{21}} + {\alpha _{22}} + {\lambda _2}{\alpha _{21}}\; {\alpha _{22}}$$

and if *λ*_*2*_ = 0 the necessary condition is:

(24) $${\beta _r} - {\mu _r} > {\alpha _{21}} + {\alpha _{22}}.$$

In this scenario, assuming mutants have an average reproduction rate greater than one and the reproductive capacity of wildtype bacteria is lower than that of mutants, only mutants survive while wildtype bacteria go extinct.

Finally, if *R*_*s*_ *> 1* and *R*_*s*_ *> R*_*r*_, the equilibrium point *P*_*2*_ is within *Ω* and is both locally and asymptotically stable. Here, when wildtype bacteria have a reproduction rate greater than one and a higher reproductive capacity than mutants, both strains can coexist. Despite the lower reproductive capacity of mutants compared to wildtype bacteria, the occurrence of spontaneous mutations in wildtype strains enables their survival.

## Results

### Numerical simulations

This section gives some numerical justification for the equilibrium points and their stability criterion. Since these equilibrium points demonstrate the free-infection, all-mutant, and coexistent of wildtype and mutant bacteria conditions for bacteria population, it is important to have some numerical justification for them. The parameters used in the simulations are constant and are given in Table 1. For the numerical simulation, we consider an individual with a disease caused by *Staphylococcus aureus* bacteria that develop resistance to antibiotics *M* and *N* through mutation. The antibiotic interaction parameter for wildtype and mutant bacteria are *(λ*_*1*_*)* and *(λ*_*2*_*)*, respectively. As underlined in the work of Torella et al. (2010), *λ* equals $0$ for additive interaction, 1 for synergistic interaction, and −1 for antagonistic interaction. Our simulation follows three scenarios. In the first scenario, antibiotics interact additively against wildtype and the mutant bacteria (*λ*_*1*_ *= λ*_*2*_ = 0). In the second scenario, antibiotics interact synergistically with the wildtype bacteria but interact antagonistically with mutants (*λ*_*1*_ *= 1, λ*_*2*_ = −1). In the third scenario, antibiotics interact antagonistically with wildtype bacteria but synergistically with mutants (*λ*_*1*_ *= −1, λ*_*2*_ = 1). Here, for the sake of simplicity, we assume that antibiotics *M* and *N* have the same maximum kill rate *(E*_*max*_*)* on wildtype bacteria (see Table 1). However, the maximum kill rate of both antibiotics declined against mutants (see Table 1) even though both same. We also assume that the *IC*_*50*_’s of $M$ and $N$ antibiotics are the same. Nevertheless, this can be achieved by a simple change of variables, scaling by an appropirate value.

**Table 1:** Interpretation and considered values of the parameters for the model (5).

Parameter	Description	Value	Units	Ref.
$K$	Bacteria carrying capacity	${10^9}$	Cells	Michel et al. (2008)
${\beta _s}$	The growth rate of sensitive bacteria	$1$	${h^{ - 1}}$	Michel et al. (2008)
${\beta _r}$	The growth rate of resistant bacteria	$0.65$	${h^{ - 1}}$	Michel et al. (2008)
${\mu _s}$	The natural death rate of sensitive bacteria	$0.5$	${h^{ - 1}}$	Michel et al. (2008)
${\mu _r}$	The natural death rate of resistant bacteria	$0.5$	${h^{ - 1}}$	Michel et al. (2008)
$m$	The mutation rate of sensitive bacteria	${10^{ - 8}} + {10^{ - 6}}$	$mut \times gen$	Touat et al. (2021)
$E_{max}^{M,S}$	The maximal kill rate of sensitive bacteria with the antibiotic *M*	1.5	${h^{ - 1}}$	Touat et al. (2021)
$E_{max}^{N,S}$	The maximal kill rate of sensitive bacteria with the antibiotic *N*	1.5	${h^{ - 1}}$	Hypothesis
$E_{max}^{M,R}$	The maximal kill rate of resistant bacteria with the antibiotic *M*	1.1	${h^{ - 1}}$	Michel et al. (2008)
$E_{max}^{N,R}$	The maximal kill rate of resistant bacteria with the antibiotic *N*	1.1	${h^{ - 1}}$	Hypothesis
$IC_{50}^{M,S}$	The concentration of the antibiotic *M*, which has a half-maximum effect on sensitive bacteria	0.25	$\mu g/ml$	Michel et al. (2008)
$IC_{50}^{N,S}$	The concentration of the antibiotic *N*, which has a half-maximum effect on sensitive bacteria	0.25	$\mu {\rm g}/{\rm ml}$	Hypothesis
$IC_{50}^{M,R}$	The concentration of the antibiotic *M*, which has a half-maximum effect on resistant bacteria	5	$\mu {\rm g}/{\rm ml}$	Michel et al. (2008)
$IC_{50}^{N,R}$	The concentration of the antibiotic *N*, which has a half-maximum effect on resistant bacteria	5	$\mu {\rm g}/{\rm ml}$	Hypothesis
$\; {\theta _1}$	hourly dose of antibiotic	0.21	$mg/h$	Touat et al. (2021)
$\; {\theta _2}$	hourly dose of the antibiotic *N*	0.42	$mg/h$	Touat et al. (2021)
${\mu _1}$	The degradation rate of the antibiotic *M*	0.0025	${h^{ - 1}}$	Touat et al. (2021)
${\mu _2}$	The degradation rate of the antibiotic *N*	0.0021	${h^{ - 1}}$	Touat et al. (2021)
$\; {\lambda _{1\; }}$	Interaction parameter between the antibiotics *M* and *N* for sensitive bacteria	varies between [−1.5, 1.5]	–	Techitnutsarut & Chamchod (2021)
$\; {\lambda _{2\; }}$	Interaction parameter between the antibiotics *M* and *N* for resistant bacteria	varies between [−1.5, 1.5]	–	Techitnutsarut & Chamchod (2021)

**Note:**

The Data are deduced from the literature.

**Figure 1:**
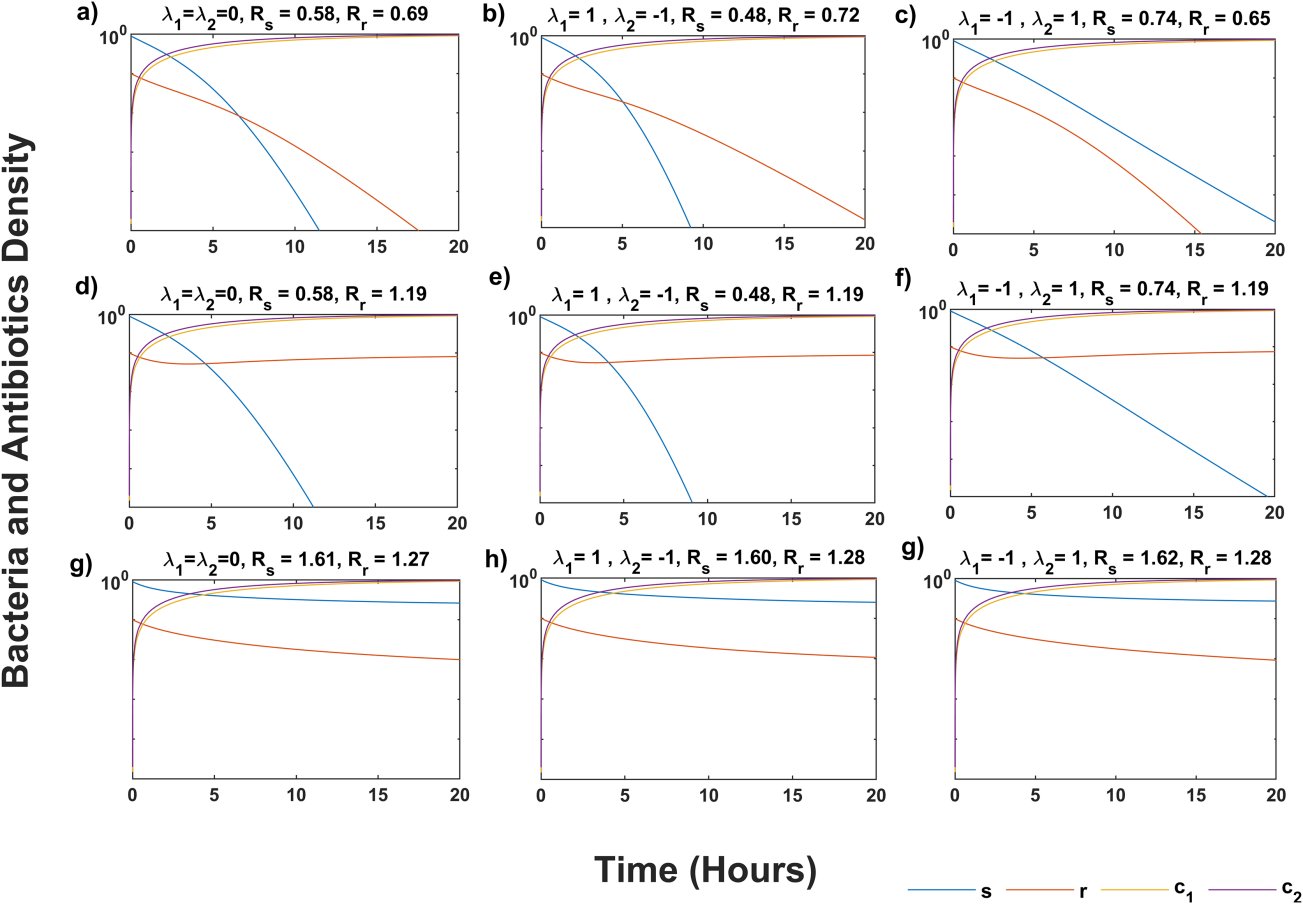
Temporal course of sensitive (s) and resistant (r) bacteria population under three scenarios of antibiotics interaction for different values of *R_s_* and *R_r_*. During the additive ( λ_1_ = λ_2_ = 0) effect of antibiotic interaction on both *s* and *r* bacteria for (A) infection-free (R_s_ < 1, R_r_ < 1), (D) all-resistance (*R_s_ < 1, R_r_ > 1*), and (G) coexistence (*R_s_ > 1, R_r_ > 1*) cases. During the synergistic ( λ_1_ = 1) effect of antibiotic interaction on the *s* bacteria, and antagonistic ( λ_2_ = −1) effect on *r* bacteria for (B) infection-free (*R_s_ < 1, R_r_ < 1*), (E) all-resistance (*R_s_ < 1, R_r_ > 1*), and (H) coexistence (*R_s_ > 1, R_r_ > 1*) cases. During the antagonistic ( λ_1_ = −1) effect of antibiotic interaction on the *s*, and synergistic ( λ_2_ = 1) effect on the *r* bacteria for (C) infection-free (*R_s_ < 1, R_r_ < 1*), (F) all-resistance (*R_s_ < 1, R_r_ > 1*), and (i) coexistence (R*_s_ > 1, R_r_ > 1*) cases. Here *c_1_* and *c_2_* are the concentration of antibiotics, M and N, respectively. Simulations are done using parameter values in Table 1 and bacteria and antibiotic concentration (y-axis) given in the log plot. The solution of system (5) approaches *P_0_* in (A–C), *P_1_* in (D–F), and *P_2_* in (G–I).

Figure 1 shows that the system (5) solution converges to the infection-free equilibrium *P*_*0*_, as indicated by *R*_*s*_ *< 1* and *R*_*r*_ *< 1* in all scenarios (Figs. 1A–1C). Synergistic antibiotic interaction results in lower reproductive numbers for both wildtype and mutant bacteria compared to additive interaction. Conversely, antagonistic interaction leads to higher reproductive numbers. In Figs. 1D–1F, where *R*_*s*_ *< R*_*r*_ and *R*_*r*_ *> 1*, the solutions approach the equilibrium point *P*_*1*_, indicating mutant bacteria evasion. In Figs. 1G–1I, where *R*_*s*_ *> R*_*r*_ and *R*_*s*_ *> 1*, the system (5) converges to the equilibrium point *P*_*2*_, showing stabilization of less fit mutants by mutations from wildtype bacteria.

### Impact of combination therapy on the minimum inhibitory concentration of mutants

Here we inspect the relationship between the interaction parameters of antibiotics for wild-type bacteria (*λ*_*1*_) and mutants (λ_2_) on the MIC. First, we analytically investigate the influence of (*λ*_*2*_) on the MIC of the mutants. Therefore, we take *R*_*r*_ = 1, for no visible growth, in Eq. (8):

(25) $${R_r} = \displaystyle{{{\beta _r}} \over {\left( {{\alpha _{21}}{\alpha _{22}}{\lambda _2} + \; {\alpha _{21}} + \; \; {\alpha _{22}}} \right) + {\mu _r}}} = 1.$$

Solving for (λ_2_) we get:

(26) $${\lambda _2} = \displaystyle{{{\beta _r} - {\mu _r} - {\alpha _{21}} - {\alpha _{22}}} \over {{\alpha _{21}}{\alpha _{22}}}} = \displaystyle{{{\beta _r} - {\mu _r} - \left( {\displaystyle{{E_{max}^r} \over {IC_{50}^{M,R}}}\; \displaystyle{{{\theta _1}} \over {\; {\mu _1}}}} \right) - \left( {\displaystyle{{E_{max}^r} \over {IC_{50}^{N,R}}}\; \displaystyle{{{\theta _2}} \over {\; {\mu _2}}}} \right)} \over {\left( {\displaystyle{{E_{max}^r} \over {IC_{50}^{M,R}}}\; \displaystyle{{{\theta _1}} \over {\; {\mu _1}}}} \right)\; \; \; \left( {\displaystyle{{E_{max}^r} \over {IC_{50}^{N,R}}}\; \displaystyle{{{\theta _2}} \over {\; {\mu _2}}}} \right)}}.$$

For the simplicity, we assume that both antibiotics have the same minimal inhibitory concentration ( *IC*^*M,R*^_*50*_ = *IC*^*N,R*^_*50*_ = *IC*^*R*^_*50*_), which can also be obtained by changing the variables and maximum killing rate (*E*^*M,R*^_*max*_ = *E*^*N,R*^_*max*_ = *E*^*R*^_*max*_) for mutants. Here we investigate only the condition where *μ*_*1*_ *= μ*_*2*_ and *θ*_*1*_ *= θ*_*2*_. Then, Eq. (26) is written as:

(27) $${\lambda _2} = \displaystyle{{{\beta _r} - {\mu _r} - 2\left( {\displaystyle{{E_{max}^r} \over {IC_{50}^R}}\; \displaystyle{{{\theta _1}} \over {\; {\mu _1}}}} \right)} \over {{{\left( {\displaystyle{{E_{max}^r} \over {IC_{50}^R}}\; \displaystyle{{{\theta _1}} \over {\; {\mu _1}}}} \right)}^2}}}.$$

We have found (see Supplemental Information equation S26–S30):

(28) $$IC_{50}^R = \displaystyle{{E_{max}^r\; MI{C_r}} \over {{\beta _r} - {\mu _r}}}$$

where *MIC*_*r*_ is the MIC for mutants treated with a single antibiotic. Substituting Eqs. (28) to (27), we reach

(29) $${\lambda _2} = \displaystyle{{MI{C_r}^2{\theta _1}^2 - 2\; MI{C_r}{\mu _1}{\theta _1}} \over {\left( {\; ({\beta _r} - {\mu _r}} \right)\; {\mu _1}{)^2}}}.$$

**Figure 2:**
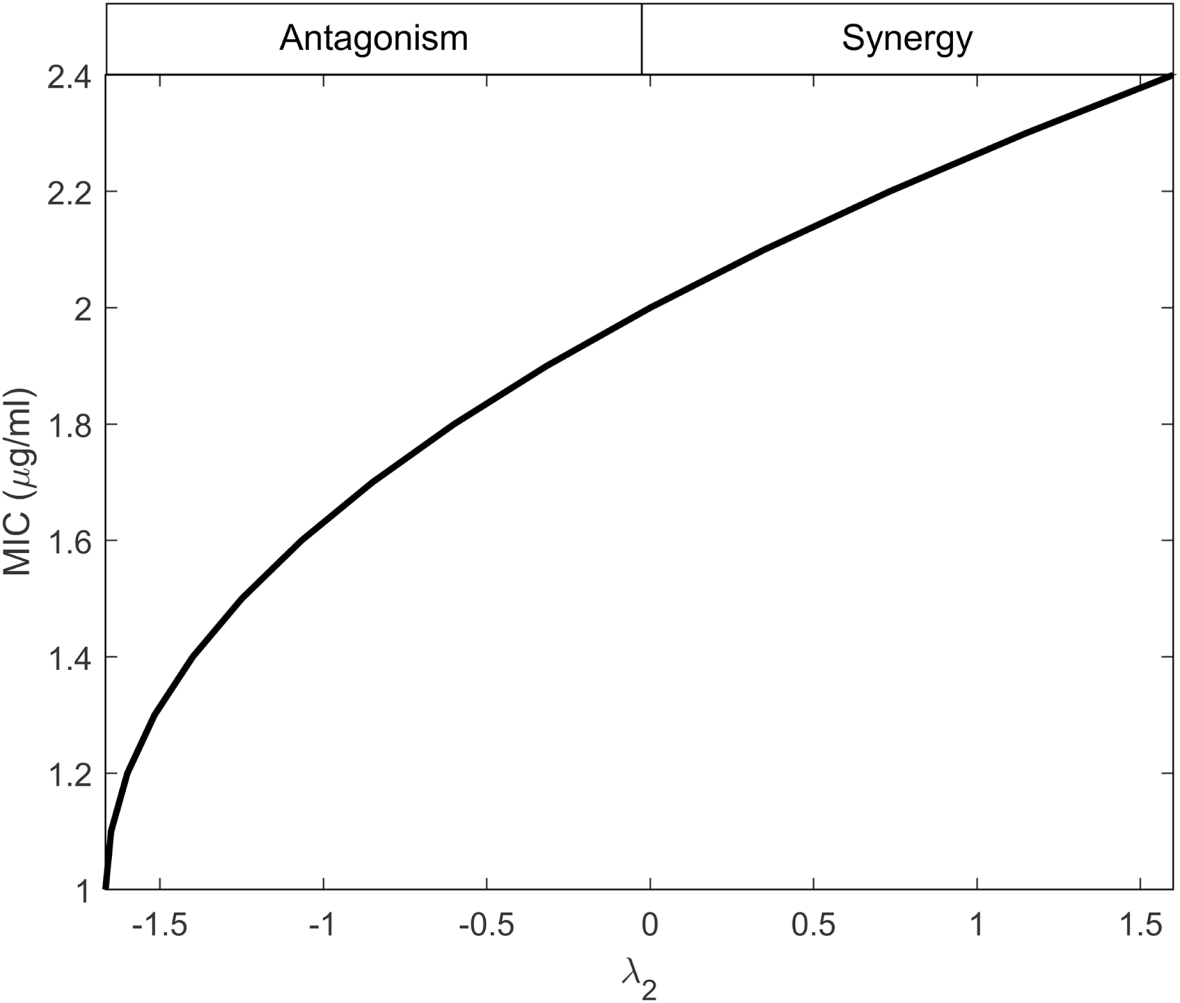
Minimum inhibitory concentration (MIC) of resistant bacteria as a result of antibiotic interaction. Here, the y-axis displays the equivalent MIC while the x-axis displays the intensity of the interaction of antibiotics (*λ_2_*) against resistant bacteria. The synergism and antagonism proxies are respectively when *λ_2_ > 0* and *λ_2_ < 0*.

Equation (29) establishes a connection between *λ*_*2*_ and *MIC*_*r*_, indicating that λ_2_ depends on the minimum effectiveness of antibiotics when used individually, as reflected by the maximum MIC value. This behavior is illustrated in Fig. 2, considering the values provided in Table 1. The figure demonstrates that as the interactions shift from antagonistic to synergistic, the antibiotics can have higher MIC values. Synergistic interactions compensate for the inefficiency of a single antibiotic, whereas antagonistic interactions require the antibiotics to be highly effective when used individually.

### Linking antibiotic interaction to the growth rate of the mutants which capture resistance

Antagonistic interactions benefit both wildtype bacteria and mutants, prompting us to explore their evolutionary implications. By comparing the growth rates of wildtype and mutant bacteria, we can determine the dominant population (Kotil & Vetsigian, 2018). Those with a growth advantage can exert control over the gene pool, rapidly passing on their advantageous qualities to future generations. This enables the population to expedite its development when beneficial traits, such as resistance, spread and the bacteria adapt to capitalize on this advantage.

Now we suppose a quasi-stable condition for concentrations, and we compute the maximum growth rate of wildtype and mutant bacteria when *s* and *r* are close to zero in system (5). To calculate the growth rate of wildtype and mutant bacteria, we divide the Eqs. (5a) and (5b) with *s* and *r*, respectively. We reach

(30a) $${G_s} = {\beta _s} - m - \left( {\left( {{\alpha _{11}}{\alpha _{12}}{\lambda _1} + \; {\alpha _{11}} + \; \; {\alpha _{12}}} \right) + {\mu _s}} \right)$$

(30b) $${G_r} = {\beta _r} - \left( {({\alpha _{21}}{\alpha _{22}}{\lambda _2} + \; {\alpha _{21}} + \; \; {\alpha _{22}}} \right) + {\mu _r})$$

where *G*_*s*_ and *G*_*r*_ are the growth rates of wildtype and mutant bacteria in quasi-stable conditions, respectively. To simplify the analysis, we assume that both antibiotics have the same minimal inhibitory concentration for mutant bacteria (*IC*^*M,R*^_*50*_ = *IC*^*N,R*^_*50*_ = *IC*^*R*^_*50*_) and wildtype bacteria (*IC*^*M,S*^_*50*_ = *IC*^*N,S*^_*50*_ = *IC*^*S*^_*50*_). We also assume that both antibiotics have the same maximum kill rate for mutant (*E*^*M,R*^_*max*_ = *E*^*N,R*^_*max*_ = *E*^*R*^_*max*_) and wildtype (*E*^*M,S*^_*max*_ = *E*^*N,S*^_*max*_ = *E*^*S*^_*max*_) bacteria. So that

${\alpha _{11}} = \displaystyle{{E_{max}^S} \over {IC_{50}^S}}\; \displaystyle{{\; {\mu _1}} \over {{\theta _1}}}, {\alpha _{12}} = \displaystyle{{E_{max}^S} \over {IC_{50}^S}}\; \displaystyle{{\; {\mu _2}} \over {{\theta _2}}}$

and

${\alpha _{21}} = \displaystyle{{E_{max}^R} \over {IC_{50}^R}}\; {{\; {\mu _1}} \over {{\theta _1}}}, {\alpha _{22}} = {{E_{max}^R} \over {IC_{50}^R}}\; {{\; {\mu _2}} \over {{\theta _2}}}.$

Here, we will investigate only the condition where *μ*_*1*_ *= μ*_*2*_ and *θ*_*1*_ *= θ*_*2*_. Consequently, *α*_*11*_ *= α*_*12*_ and *α*_*21*_ *= α*_*22*_ then Eqs. (30a) and (30b) has become

(31a) $${G_s} = {\beta _s} - m - \left( {\left( {{\alpha _{11}}^2\; {\lambda _1} + 2\; {\alpha _{11}}} \right) + {\mu _s}} \right)$$

(31b) $${G_r} = {\beta _r} - \left( {({\alpha _{21}}^2{\lambda _2} + 2\; {\alpha _{21}}} \right) + {\mu _r}).$$

Since antibiotics have less effect on resistant mutant bacteria than wildtype, so we can write *α*_*11*_ *= δ α*_*12*_ for some *δϵR*, substituting this in the Eq. (31b) we get:

(32) $${G_r} = {\beta _r} - \left( {{\delta ^2}\; {\alpha _{11}}^2{\lambda _2} + 2\; \delta \; {\alpha _{11}}} \right) + {\mu _r}).$$

Solving the Eq. (31a) for *α*_*11*_ we find

(33) $${\alpha _{11}} = \displaystyle{{ - 1 + \sqrt {\; - {\lambda _{1\; }}{\mu _s} + \; {\lambda _{1\; }}{\beta _s}\; - {\lambda _{1\; }}{G_s}\; - {\lambda _{1\; }}m + 1} } \over {\; {\lambda _{1\; }}}}$$

substituting the Eq. (33) into the Eq. (32)

(34) $$\eqalign{
  & {G_r} = {\beta _r} - {{{\delta ^2}\;{{\left( { - 1 + \sqrt {\; - {\lambda _{1\;}}{\mu _s} + \;{\lambda _{1\;}}{\beta _s}\; - {\lambda _{1\;}}{G_s}\; - {\lambda _{1\;}}m + 1} } \right)}^2}\;{\lambda _2}} \over {\;{\lambda _{1\;}}^2}}  \cr 
  & \,\,\,\,\,\,\,\,\,\,\,\,\,\,\,\, - \;{{2\;\delta \;\left( { - 1 + \sqrt {\; - {\lambda _{1\;}}{\mu _s} + \;{\lambda _{1\;}}{\beta _s}\; - {\lambda _{1\;}}{G_s}\; - {\lambda _{1\;}}m + 1} } \right)} \over {\;{\lambda _{1\;}}}} - {\mu _r}). \cr} $$

**Figure 3:**
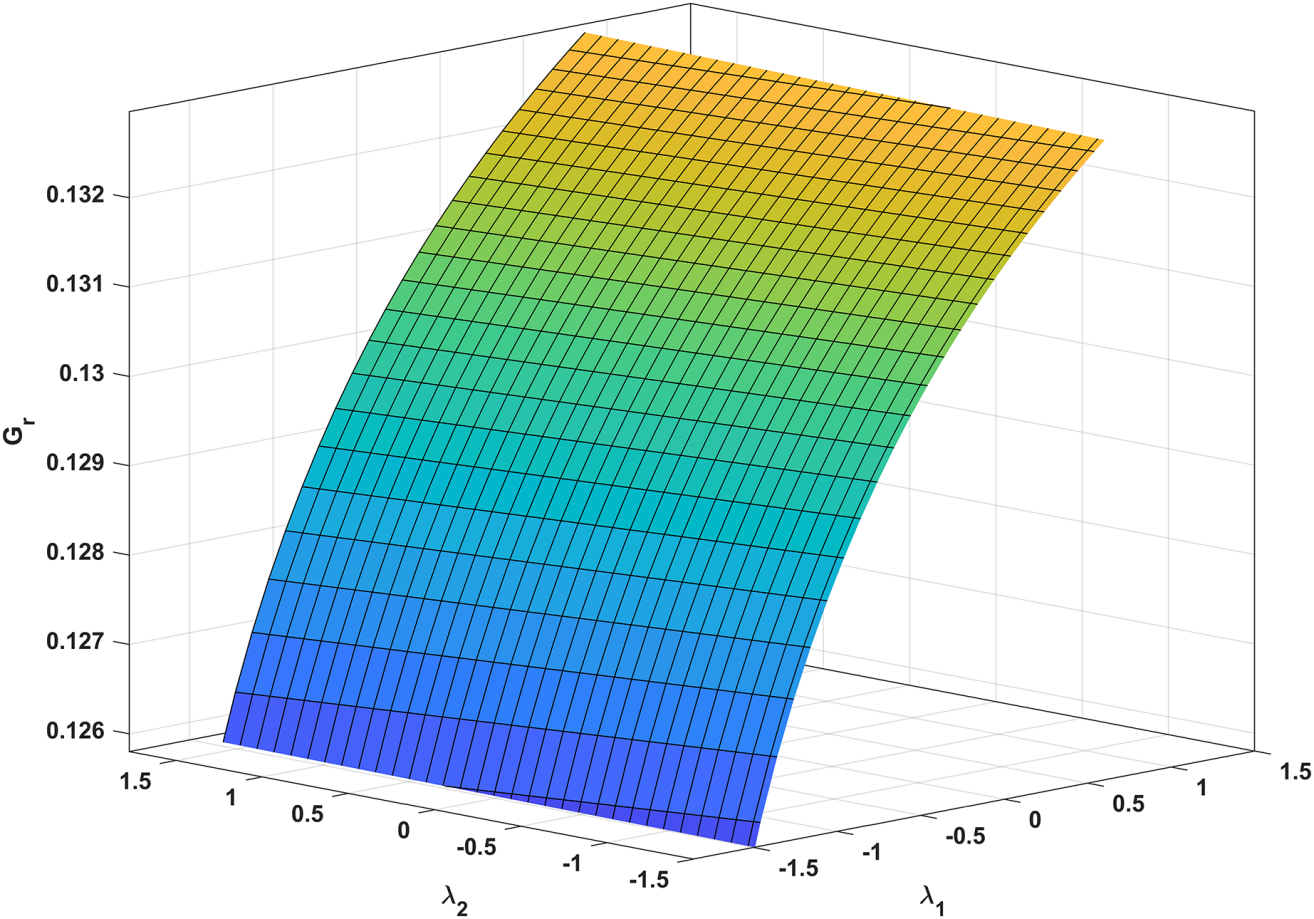
Correlation between antibiotic interaction level and growth rate of resistant strains. The x-axis and y-axis in this graph show the level of antibiotic interaction with sensitive (*λ1*) and resistant (*λ2*) bacteria, respectively, while the z-axis shows the equivalent growth rate (*Gr*) of resistant strains. The synergism and antagonism proxies are respectively when *λ1, λ2 > 0* and *λ1, λ2 < 0*.

**Figure 4:**
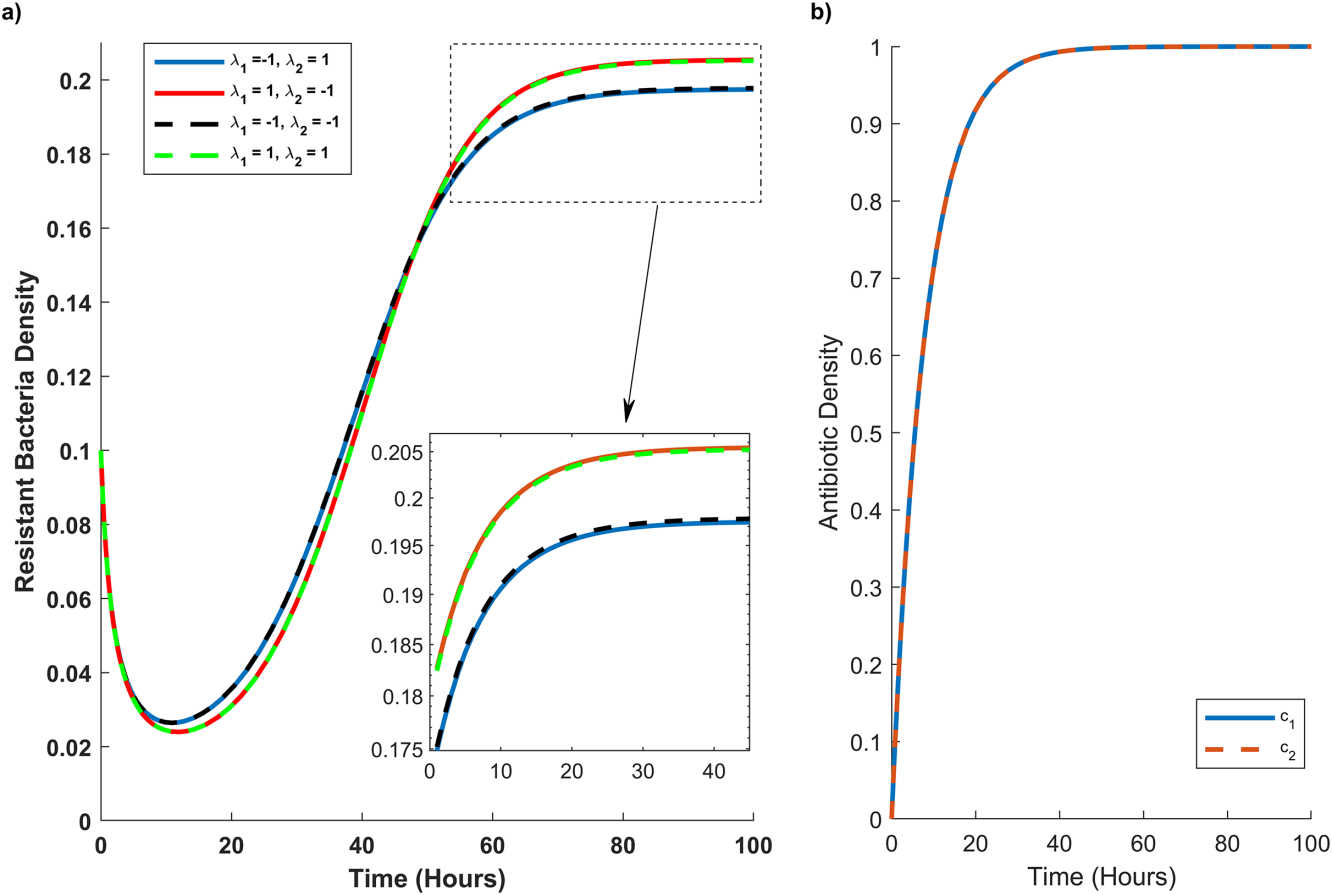
Temporal course of resistant (r) bacteria population under different combination scenarios. (A) Resistant bacteria population over time. (B) Antibiotic *M* (blue line) and *N* (red dash line) concentration (*c_1_*) and (*c_2_*), respectively, over time. In graph (A) blue line reveals the synergistic (*λ_1_ = 1*) effect of *M* and *N* antibiotics on sensitive bacteria and the antagonistic (*λ_2_ = −1*) effect of *M* and *N* antibiotics on resistant bacteria. The red line illustrates the antagonistic (*λ_1_ = −1*) effect of *M* and *N* antibiotics on sensitive bacteria and the synergistic (*λ_2_ = 1*) effect of *M* and *N* antibiotics on resistant bacteria. The black line shows the antagonistic (*λ_1_ = −1, λ_2_ = −1*) effect of *M* and *N* antibiotics on sensitive and resistant bacteria. The green line synergistic (*λ_1_ = 1, λ_2_ = 1*) effect of *M* and *N* antibiotics on sensitive and resistant bacteria. The rectangular dash point out the resistant bacteria population when the concentration of *M* and *N* antibiotics are at their maximum level (*c_1_ = c_2_ = 1*).

In Fig. 3, we analyze the growth rate surface (*G*_*r*_) of resistance-acquiring mutants by plotting it against antibiotic interaction parameters (*λ*_*1*_ and *λ*_*2*_) for wildtype and mutant bacteria. The parameter values from Table 1 are used, and *G*_*s*_ is set to 0 to represent complete inhibition of wildtype bacteria. Figure 3 confirms our expectations by demonstrating that *G*_*r*_ decreases with increasing *λ*_*1*_ antagonism in wildtype bacteria and increases with increasing *λ*_*2*_ antagonism in mutant bacteria. Notably, *G*_*r*_ is more influenced by *λ*_*1*_ than *λ*_*2*_. Additionally, a numerical simulation in Fig. 4 validates our analytical findings from Fig. 3.

In Fig. 4A, we simulate the temporal progression of wildtype and mutant strains under four antibiotic combination therapies. Firstly, antibiotics *M* and *N* exhibit antagonistic effects on the wild type and synergistic effects on the mutant. Secondly, their combination impacts the mutant antagonistically and the wild type synergistically. Thirdly, they have antagonistic effects on both mutant and wild-type cells. Ultimately, antibiotics *M* and *N* demonstrate synergistic effects on both cell types.

Figure 4A reveals that mutant bacteria reach their highest population density at *λ*_*1*_ *= 1* (synergistic). Despite the antagonistic interactions between antibiotic pairs for mutant bacteria (*λ*_*2*_ *= −1*), the mutant population acquiring resistance is lower when antibiotic concentrations are low in the second and third combination therapies. As the antibiotic concentration approaches its maximum, the population decreases in the same combination therapy scenario. Consequently, Fig. 4A indicates that *λ*_*2*_ has a minimal impact on the growth rate of mutant bacteria acquiring resistance.

## Discussion

The rapid spread of antibiotic-resistant pathogens has driven the use of antibiotic combinations to maintain efficacy and combat resistance. In this study, we developed a mathematical model to analyze the population dynamics of wildtype and mutant bacteria under interacting antibiotics. The model evaluates the relationship between antibiotics for wildtype and mutant bacteria, including their synergistic and antagonistic interactions, on the growth rate of mutant strains which acquire resistance. Stability analysis examined equilibrium states with infection-free, all-mutant, and coexistence of wildtype and mutant bacteria. Additionally, numerical simulations showcased the temporal dynamics of wildtype and mutant bacteria under different combination therapies.

Clinics often employ synergistic antibiotic combinations for enhanced efficacy at lower doses and reduced toxicity (Lv et al., 2022; Yilancioglu, 2019). However, these combinations also accelerate the evolution of antibiotic resistance (Hegreness et al., 2008; Yilancioglu, 2019), providing a gateway for the selective advantage of resistance mutations. Our findings align with these conclusions, as supported by our analytic and numeric results (Eq. (34) and Fig. 3, respectively). Notably, Fig. 3 demonstrates that increasing the synergistic level between antibiotics boosts the growth rate of resistant strains.

On the other hand, antagonistic antibiotic combinations effectively prevent antibiotic resistance in wildtype bacteria (Chait, Craney & Kishony, 2007), making them recommended as combination therapy despite lower efficacy at higher doses (Hegreness et al., 2008; Torella et al., 2010). However, during combination therapy, the synergistic interaction between antibiotics and wildtype bacteria evolves into antagonistic interaction within the same population (Pena-Miller et al., 2013). The intricate nature of antibiotic interactions allows synergy to be lost or flipped for reasons other than competitive release (Pena-Miller et al., 2013; Palmer, Angelino & Kishony, 2010). Over time, synergy deteriorates due to selection for antibiotic-resistant alleles, but it can be reversed when antibiotics break down into non-antibiotic metabolites (Palmer, Angelino & Kishony, 2010). This suggests that while antibiotics select for resistant strains, other natural processes may exist that counteract resistance, resulting in the coexistence of resistant mutant and wildtype bacterial strains (D’Costa et al., 2006). We found that antagonistic interactions against wildtype bacteria play a crucial role in reducing the rate at which resistant mutant bacteria proliferate through mutation, while the antagonistic interaction against mutant bacteria only minimally accelerates evolution.

Furthermore, Torella et al. (2010) discovered that synergistic interactions reduce the clearing time for susceptible wildtype bacteria while enhancing the competitive advantage of resistant mutant bacteria. Conversely, antagonistic interactions prolong the purification time and diminish the competitive advantage of antibiotic-resistant mutants. Our findings in Fig. 3 demonstrate that mutants exposed to antagonistic antibiotics outperform resistant mutant strains.

Eventually, our numerical simulations were based on the parameters of *Staphylococcus aureus* (Techitnutsarut & Chamchod, 2021), but our analytic results are not limited to specific antibiotics or bacteria. Any two antibiotics of the same class, targeting the same site, can lead to similar outcomes if bacteria develop resistance by mutating the target and gain a fitness advantage. For example, methicillin-resistant *S. aureus* (MRSA) poses a significant and persistent risk to human health (Panchal et al., 2020; Chuang & Huang, 2013; Chu et al., 2005). MRSA acquires resistance to multiple β-Lactam antibiotics (Panchal et al., 2020) by producing a non-native penicillin binding protein 2A (PBP2A) encoded by mecA (Panchal et al., 2020; Pinho, de Lencastre & Tomasz, 2001). Mutations in the mecI gene (a repressor of mecA) enable MRSA to thrive in the presence of β-Lactam antibiotics by producing PBP2A (Oliveira & de Lencastre, 2011).

## Conclusion

This article has investigated the relationship between the synergistic and antagonistic interaction of antibiotics for wild-type and mutant bacteria on the growth rate of resistant mutant strains. In this direction, a deterministic model has been developed to achieve the goal. The effective reproduction number, the growth rate of resistant strains, and antibiotic-antibiotic interaction have been demonstrated analytically. Moreover, the condition for free-bacteria, all mutant-bacteria, and coexistence equilibrium points have been determined.

The theoretical findings have been successfully supported by numerical analysis. The main findings of this work are as follows:

- We have clarified that antagonism against the wildtype bacteria has a more critical role than synergistic in slowing the growth rate of mutant bacteria. In contrast, the antagonistic interaction in the mutant type speeds up evolution but minimally.
- Our analytical results suggest that it would be more appropriate to develop combine therapy strategy against wildtype bacteria as opposed to mutant bacteria in order to slow down the acquisition of antibiotic resistance rather than stop the development of resistant strains. It has been revealed that the best multidrug therapy that can stand the test of time must include highly effective antibiotics that interact antagonistically with wildtype bacteria, if possible, interact synergistically with the mutant bacteria. The potential impact of our finding is that this kind of therapy slow down the acquisition of resistance.

## Supplemental Information

10.7717/peerj.16917/supp-1 Supplemental Information 1 Supplemental Material.

## Additional Information and Declarations

### Data Availability

The following information was supplied regarding data availability: The datasets are available at GitHub and Zenodo: - https://github.com/raminnashebi/Mathematical-Modelling-of-Drug-Interaction-on-Evolution-of-Antibiotic-Resistance.git. - Ramin Nashebi, Murat Sari, & Seyfullah Kotil. (2023). Mathematical Modelling of antibiotic Interaction on Evolution of Antibiotic Resistance: An Analytical Approach. Zenodo. https://doi.org/10.5281/zenodo.8337142.
