# Supplementary material for: Mathematical modelling of antibiotic interaction on evolution of antibiotic resistance: an analytical approach
Source: PeerJ. 2024 Feb 26;12:e16917. doi: 10.7717/peerj.16917 (PMC10903357; doi:10.7717/peerj.16917)
Supplement: Supplemental Information 1 [file peerj-12-16917-s001.docx]

Mathematical Modelling of antibiotic Interaction on Evolution of Antibiotic Resistance: An Analytical Approach

Ramin Nashebi^1^, Murat Sari^2^, Seyfullah Kotil^3^

^1^ Department of Mathematics, Yildiz Technical University, Istanbul, Turkey,

^2^ Department of Mathematical Engineering, Istanbul Technical University, Istanbul, Turkey

^3^ Department of Biophysics, Bahcesehir University, Medical School, Istanbul, Turkey

^4^ Department of Molecular Biology and Genetics, Faculty of Arts and Sciences, Bogazici University, Istanbul, Turkey

Corresponding Author:

Seyfullah Kotil^2^

Rumeli Hisarı, Hisar Ustu Nispetiye Cd No:7, 34342 Sariyer/ Department of Molecular Biology and Genetics, Faculty of Arts and Sciences/ Bogazici University/ Istanbul/ Turkey

Email address: enesseyfullah.kotil@boun.edu.tr

**Derivation of Equilibrium Points**

The equilibria of system (5) are given by the solutions of the system of algebraic equations:

| $\beta_{s}s\left( 1-(s+r) \right)-{(q}_{1}c_{1}+q_{2}c_{2})s-\left( (\alpha_{11}c_{1}+\alpha_{12}c_{2}+\lambda_{1}\alpha_{11} \alpha_{12}c_{1}c_{2})+\mu_{s} \right)s=0$ | (S1a) |
| --- | --- |
| $\beta_{r}r\left( 1-(s+r) \right)+{(q}_{1}c_{1}+q_{2}c_{2})s-\left( (\alpha_{21}c_{1}+\alpha_{22}c_{2}+\lambda_{2}\alpha_{21} \alpha_{22}c_{1}c_{2})+\mu_{r} \right)r=0$ | (S1b) |
| $\mu_{1}-\mu_{1}c_{1}=0$ | (S1c) |
| $\mu_{2}-\mu_{2}c_{2}=0$. | (S1d) |

From the equations (S1c) and (S1d), we have *c_1_ = c_2_ = 1*. Replacing *c_1_* and *c_2_*  in the equations (S1a) and (S1b), we obtain:

| $\beta_{s}s\left( 1-(s+r) \right)-{(q}_{1}+q_{2})s-\left( (\alpha_{11}+\alpha_{12}+\lambda_{1}\alpha_{11} \alpha_{12})+\mu_{s} \right)s=0$ | (S2a) |
| --- | --- |
| $\beta_{r}r\left( 1-(s+r) \right)+{(q}_{1}+q_{2})s-\left( (\alpha_{21}+\alpha_{22}+\lambda_{2}\alpha_{21} \alpha_{22})+\mu_{r} \right)r=0$. | (S2b) |

It holds from the equation (S2a) that *s=0* or:

| $\beta_{s}\left( 1-(s+r) \right)-m-\left( \left( \alpha_{11}+\alpha_{12}+\lambda_{1}\alpha_{11} \alpha_{12} \right)+\mu_{s} \right) =0$ | (S3) |
| --- | --- |

where *m=q_1_+q_2._*

Assume *s=0* replacing this value in the equation (S2b) we obtain:

| $\beta_{r}r-\beta_{r}r^{2}-\left( (\alpha_{21}+\alpha_{22}+\lambda_{2}\alpha_{21} \alpha_{22})+\mu_{r} \right)r=0$ | (S4) |
| --- | --- |

which implies $r=0$ or:

| $r=\frac{R_{r}-1}{R_{r}}$ | (S5) |
| --- | --- |

where

| $R_{r}=\frac{\beta_{r}}{(\alpha_{21}\alpha_{22}\lambda_{2}+ \alpha_{21}+ \alpha_{22})+\mu_{r}}$. | (S6) |
| --- | --- |

Therefore, we obtain the equilibrium solutions

| $P_{0}=(0,0,1,1)$ | (S7a) |
| --- | --- |
| $P_{1}=\left( 0,\frac{R_{r}-1}{R_{r}},1,1 \right)$. | (S7b) |

From equation (S5), it follows that a necessary and sufficient condition for the biological sense of *P_1_* is *R_r_ >1*. Now, for *s≠0* the equation (S2a) is reduced to:

| $s=\frac{R_{s}-1}{R_{s}}-r$ | (S8) |
| --- | --- |

where

| $R_{s}=\frac{\beta_{s}}{m+\left( \alpha_{11}+\alpha_{12}+\lambda_{1}\alpha_{11} \alpha_{12} \right)+\mu_{s}}$. | (S9) |
| --- | --- |

From equation (S8), it is concluded that a necessary condition for the existence of sensitive and resistant bacteria is *R_s_ >1* Also, a sufficient condition for $s$ to be positive is:

| $\frac{R_{s}-1}{R_{s}}>r$. | (S10) |
| --- | --- |

Substituting equation (S8) in the equation (S2b) and solving for *r* we obtain:

| $r=\frac{m\left( \frac{R_{s}-1}{R_{s}} \right)}{\beta_{r}\left( \frac{1}{R_{r}}-\frac{1}{R_{s}} \right)+m}$. | (S11) |
| --- | --- |

Replacing $r$defined by (S11) in the inequality (S10), it is easy to verify that *s > 0* is equivalent to *R_s_ > R*_r_. Further, *r > 0* if 1/*R*_r_ > 1/ *R_s_.* Therefore, a necessary condition for *s* and *r* to be positive is *R_s_ > R*_r_.

**Stability Analysis of Equilibrium Points**

By evaluating the equation (14) Jacobian *J* in *P_0_* we obtain:

| $J\left( P_{0} \right)=\left[ \begin{matrix} j_{11}(P_{0}) & 0 & 0 & 0 \\ m & j_{22}(P_{0}) & 0 & 0 \\ 0 & 0 & {-\mu}_{1} & 0 \\ 0 & 0 & 0 & {-\mu}_{2} \end{matrix} \right]$. | (S12) |
| --- | --- |

The eigenvalues of *J(P_0_)* are given by:

| $\varphi_{1}=j_{11}(P_{0})=\beta_{s}-m-\left( \left( \alpha_{11}+\alpha_{12}+\lambda_{1}\alpha_{11}\alpha_{12} \right)+\mu_{s} \right)=\beta_{s}\left( \frac{R_{s}-1}{R_{s}} \right)$ | (S13a) |
| --- | --- |
| $\varphi_{2}=j_{22}(P_{0})=\beta_{r}-\left( (\alpha_{21}+\alpha_{22}+\lambda_{2}\alpha_{21} \alpha_{22})+\mu_{r} \right)=\beta_{r}\left( \frac{R_{r}-1}{R_{r}} \right)$ | (S13b) |
| $\varphi_{3}={-\mu}_{1}$ | (S13c) |
| $\varphi_{4}={-\mu}_{2}$. | (S13d) |

Since *φ_1_* and *φ_2_* are negative for *R_s_* < 1 and *R_r_* < 1, respectively, then *P_0_* is locally and asymptotically stable. Since *α_11_, α_12_*, *μ_s_, and β_s_* are positive; there are three conditions for *R_s_* < 1 if *λ_1_* > 0, *λ_1_* < 0, or *λ_1_* = 0. If *λ_1_* > 0, *λ_1_* < 0 the necessary condition for *R_s_* < 1 is:

| $\beta_{s}-\mu_{s}-m<\alpha_{11}+\alpha_{12}+\lambda_{1}\alpha_{11} \alpha_{12}$ |
| --- |

and if *λ_1_* = 0, the necessary condition is:

| $\beta_{s}-\mu_{s}-m<\alpha_{11}+\alpha_{12}$. |
| --- |

Analogously, since *α_21_, α_22_*, *μ_r_, and β_r_* are positive, there are three conditions for *R_r_* > 1, if *λ_2_* > 0, *λ_2_* < 0, or *λ_2_* = 0. If *λ_2_* > 0, *λ_2_* < 0 the necessary condition for *R_r_* < 1 is:

| $\beta_{r}-\mu_{r}<\alpha_{21}+\alpha_{22}+\lambda_{2}\alpha_{21} \alpha_{22}$ |
| --- |

and if *λ_2_* = 0 the necessary condition is:

| $\beta_{r}-\mu_{r}<\alpha_{21}+\alpha_{22}$. |
| --- |

Now, we determine the conditions for which the equilibrium *P_1_* is locally and asymptotically stable. To this end, let us observe that the Jacobian given in equation (14) evaluated in *P_1_* is given by:

| $J\left( P_{1} \right)=\left[ \begin{matrix} j_{11}(P_{1}) & 0 & 0 & 0 \\ -\beta_{r}\left( \frac{R_{r}-1}{R_{r}} \right)+m & j_{22}(P_{1}) & -(\lambda_{2}\alpha_{21}\alpha_{22}+\alpha_{21})\frac{R_{r}-1}{R_{r}} & -(\lambda_{2}\alpha_{21}\alpha_{22}+\alpha_{22})\frac{R_{r}-1}{R_{r}} \\ 0 & 0 & {-\mu}_{1} & 0 \\ 0 & 0 & 0 & {-\mu}_{2} \end{matrix} \right]$. | (S14) |
| --- | --- |

The eigenvalues of *J(P_1_)*are given by:

| $\omega_{1}=j_{11}(P_{1})= \beta_{s}\left( 1-\frac{R_{r}-1}{R_{r}} \right)-m-\mu_{s}-\left( \alpha_{11}+\alpha_{12}+\lambda_{1}\alpha_{11}\alpha_{12} \right)=\beta_{s}\left( \frac{1}{R_{r}}-\frac{1}{R_{s}} \right)$ | (S15a) |
| --- | --- |
| $\omega_{2}=j_{22}(P_{1})= \beta_{r}\left( 1-\frac{R_{r}-1}{R_{r}} \right)-\beta_{r}\left( \frac{R_{r}-1}{R_{r}} \right)-\mu_{r}-(\alpha_{21}+\alpha_{22}+\lambda_{2}\alpha_{21} \alpha_{22})=\beta_{r}\left( \frac{1-R_{r}}{R_{r}} \right)$ | (S15b) |
| $\omega_{3}={-\mu}_{1}$ | (S15c) |
| $\omega_{4}={-\mu}_{2}$. | (S15d) |

We see that *ω_1_* < 0 if and only if *R_r_* > *R_s_* and that *ω_2_* < 0 if and only if *R_r_* >1. Since $\alpha_{21}$, $\alpha_{21}$, $\mu_{r}$, and $\beta_{r}$ are positive, there are three conditions for *R_r_* > 1, if *λ_2_* > 0, *λ_2_* < 0, or *λ_2_* = 0. If *λ_2_* > 0, *λ_2_* < 0 the necessary condition for *R_r_* > 1 is:

| $\beta_{r}-\mu_{r}>\alpha_{21}+\alpha_{22}+\lambda_{2}\alpha_{21} \alpha_{22}$ |
| --- |

and if *λ_2_* = 0 the necessary condition is:

| $\beta_{r}-\mu_{r}>\alpha_{21}+\alpha_{22}$. |
| --- |

Now, we determine the conditions for which the equilibrium *P_2_* is locally and asymptotically stable. To this end, let us observe that the Jacobian given in equation (14) evaluated in *P_2_* is given by:

| $J\left( P_{2} \right)=\left[ \begin{matrix} j_{11}(P_{2}) & -\beta_{s}\bar{s} & -(\lambda_{1}\alpha_{11}\alpha_{12}+\alpha_{11})\bar{s} & -(\lambda_{1}\alpha_{11}\alpha_{12}+\alpha_{12})\bar{s} \\ -\beta_{r}\bar{r}+m & j_{22}(P_{2}) & -(\lambda_{2}\alpha_{21}\alpha_{22}+\alpha_{21})\bar{r} & -(\lambda_{2}\alpha_{21}\alpha_{22}+\alpha_{22})\bar{r} \\ 0 & 0 & {-\mu}_{1} & 0 \\ 0 & 0 & 0 & {-\mu}_{2} \end{matrix} \right]$ | (S16) |
| --- | --- |

where

| $j_{11}\left( P_{2} \right)=\beta_{s}\left( 1-\left( \bar{s}+\bar{r} \right) \right)-\beta_{s}\bar{s}-m-\mu_{s}-\left( \alpha_{11}+\alpha_{12}+\lambda_{1}\alpha_{11}\alpha_{12} \right)$ | (S17a) |
| --- | --- |
| $j_{22}(P_{2})=\beta_{r}\left( 1-(\bar{s}+\bar{r}) \right)-\beta_{r}\bar{r}-\mu_{r}-(\alpha_{21}+\alpha_{22}+\lambda_{2}\alpha_{21} \alpha_{22})$. | (S17b) |

From (S3), it follows:

| $j_{11}\left( P_{2} \right)=\beta_{s}\left( 1-\left( \bar{s}+\bar{r} \right) \right)-\beta_{s}\bar{s}-m-\mu_{s}-\left( \alpha_{11}+\alpha_{12}+\lambda_{1}\alpha_{11}\alpha_{12} \right)=-\beta_{s}\bar{s}$ | (S18) |
| --- | --- |

and from the equation (S2b), we have:

| $j_{22}(P_{2})=\beta_{r}\left( 1-(\bar{s}+\bar{r}) \right)-\beta_{r}\bar{r}-\mu_{r}-(\alpha_{21}+\alpha_{22}+\lambda_{2}\alpha_{21} \alpha_{22})=-\frac{1}{\bar{r}}(\beta_{r}\bar{r}+m\bar{s})$. | (S19) |
| --- | --- |

Substituting equations (S18) and (S19) in equation (S16), *J(P_2_)* becomes:

| $J\left( P_{2} \right)=\left[ \begin{matrix} -\beta_{s}\bar{s} & -\beta_{s}\bar{s} & -(\lambda_{1}\alpha_{11}\alpha_{12}+\alpha_{11})\bar{s} & -(\lambda_{1}\alpha_{11}\alpha_{12}+\alpha_{12})\bar{s} \\ -\beta_{r}\bar{r}+m & -\frac{1}{\bar{r}}(\beta_{r}\bar{r}+m\bar{s}) & -(\lambda_{2}\alpha_{21}\alpha_{22}+\alpha_{21})\bar{r} & -(\lambda_{2}\alpha_{21}\alpha_{22}+\alpha_{22})\bar{r} \\ 0 & 0 & {-\mu}_{1} & 0 \\ 0 & 0 & 0 & {-\mu}_{2} \end{matrix} \right]$. | (S20) |
| --- | --- |

The eigenvalues of *J(P_2_)*are:

| $\tau_{1}={-\mu}_{1}$ | (S21a) |
| --- | --- |
| $\tau_{2}={-\mu}_{2}$ | (S22b) |

and the eigenvalues of the matrix:

| $A=\left[ \begin{matrix} -\beta_{s}\bar{s} & -\beta_{s}\bar{s} \\ -\beta_{r}\bar{r}+m & -\frac{1}{\bar{r}}(\beta_{r}\bar{r}^{2}+m\bar{s}) \end{matrix} \right]$. | (S23) |
| --- | --- |

Since

| $Trace(A)=-\beta_{s}\bar{s}-\frac{1}{\bar{r}}(\beta_{r}\bar{r}^{2}+m\bar{s})<0$ | (S24) |
| --- | --- |

and

| $Det(A)=\frac{1}{\bar{r}}(\beta_{s} \beta_{r} \bar{s} \bar{r}^{2}+\beta_{s}m \bar{s}^{2})+\beta_{s} \beta_{r} \bar{s} \bar{r} - \beta_{s} \bar{s} m >0$. | (S25) |
| --- | --- |

The eigenvalues of *A* have a negative real part.

**Half maximal inhibitory concentration for resistant bacteria (*IC^R^ _50_*) as function of minimum inhibitory concentration for resistant bacteria (*MIC_r_*)**

Supposed that a single antibiotic inhibits resistant bacteria, and sensitive bacteria do not spontaneously mutate to become resistant. By underestimating the logistic growth of resistant bacteria, the equation (4b) became:

| $\frac{dR}{dt}=\beta_{r}R-\frac{E_{max}^{r}}{{IC}_{50}^{R}}CR-\mu_{r}R$ | (S26) |
| --- | --- |

Here *C* represents a concentration of antibiotics. At MIC (minimum inhibitory concentration) level *dR/dt* must equal to zero, so we can write the above equation as:

| ${0=\beta}_{r}R-\frac{E_{max}^{r}}{{IC}_{50}^{R}}CR-\mu_{r}R$ | (S27) |
| --- | --- |

Dividing both side by *R* we obtain:

| ${0=\beta}_{r}-\frac{E_{max}^{r}}{{IC}_{50}^{R}}C-\mu_{r}$ | (S28) |
| --- | --- |

Solving for *C*, we get:

| $C=\frac{{(\beta}_{r}-\mu_{r}){IC}_{50}^{R}}{E_{max}^{r}}=MIC$ | (S29) |
| --- | --- |

Solving for *IC^R^ _50_* , we get:

| ${IC}_{50}^{R}=\frac{E_{max}^{r}\cdot MIC}{{(\beta}_{r}-\mu_{r})}$ | (S30) |
| --- | --- |

**Role of synergistic interactions against wildtype bacteria and mutants on the deacceleration of antimicrobial resistance**

Our findings specifically emphasize that synergistic antibiotic interactions against wildtype bacteria do not play a pivotal role in retarding the growth rate of resistant mutants. Conversely, it is observed that when synergistic antibiotic interactions against mutants collaborate with antagonistic interactions against wildtype bacteria, there is a significant deceleration in the growth rate of resistant mutants.
